# Double Valve Replacement in a Patient With Hunter Syndrome

**DOI:** 10.7759/cureus.28961

**Published:** 2022-09-08

**Authors:** Suresh Keshavamurthy, Andra Duncan, Akshay Kumar, Carlos Trombetta, Rene Rodriguez, Carmela Tan, Eric Roselli

**Affiliations:** 1 Cardiothoracic Surgery, University of Kentucky, Lexington, USA; 2 Anesthesiology and Perioperative Medicine, Cleveland Clinic Foundation, Cleveland, USA; 3 Cardiothoracic Surgery, Cleveland Clinic Foundation, Cleveland, USA; 4 Pathology, Cleveland Clinic Foundation, Cleveland, USA

**Keywords:** glycosaminoglycans, mitral valve replacement, aortic valve replacement, mucopolysaccharidosis, hunter syndrome

## Abstract

Hunter syndrome is a rare disorder in which affected patients have significant airway abnormalities (macroglossia, small mouth opening, and supraglottic narrowing) that complicate their management. Deposition of glycosaminoglycans in the heart leads to cardiomyopathy, and cardiac valve dysplasia that can lead to valvular stenosis or regurgitation or both, necessitating valve replacement. Management of patients with Hunter syndrome is complex and needs a multidisciplinary team approach. Mechanical valve replacement is a suitable treatment option.

## Introduction

Hunter syndrome is a rare, X-linked mucopolysaccharidosis (MPS) II disorder caused by the deficiency of iduronate-2-sulfatase [1]. Affected patients have significant airway abnormalities that complicate their management. The presentation can vary between the two extremes (severe and attenuated), and most are often diagnosed between the ages of four and eight years. Oropharyngeal and tracheobronchial deposition of glycosaminoglycans (GAGs) leads to severe airway obstruction due to macroglossia, supraglottic narrowing, and tracheomalacia [2-6]. This obstructive anatomy and physiology lead to sleep apnea and airway obstruction. As the disease progresses, the respiratory system is further compromised by pulmonary restriction secondary to the effects of the disease on the thoracic skeleton. Varying degrees of hearing impairment, both conductive and sensorineural in origin, have been reported in Hunter syndrome [7,8]. Deposition of GAGs in the heart, liver, and spleen leads to cardiomyopathy, thickening and stiffening of the valve leaflets commonly leading to mitral and aortic regurgitation and/or stenosis, and hepatosplenomegaly. Bone and joint involvement results in severe skeletal deformities and limitations of joint mobility [9]. We report the preoperative preparation, and surgical and anesthetic management of an adult patient with Hunter syndrome who presented for surgical management of his valve disease.

## Case presentation

A 55-year-old male with a history of Hunter's syndrome, sleep apnea, cervical laminectomy, and gastroesophageal reflux disease (GERD) presented for evaluation of his aortic valve disease. He had undergone a cervical laminectomy (C1-3) for numbness in the neck, arms, and legs a year earlier. He also had a hearing aid for hearing impairment. Over the previous several months, he had experienced increased shortness of breath with exertion and decreased endurance after moderate exercise. An echocardiogram performed at another hospital reported an aortic valve area (AVA) of 0.6cm2 and a mean transvalvular gradient of 53 mmHg. A left heart catheterization demonstrated normal coronary arteries and severe aortic stenosis. The right heart catheterization revealed right ventricle (RV) 35/8, pulmonary artery (PA) mean pressures 26, and normal cardiac output. An aortic valve replacement (AVR) was recommended and due to his complex medical issues, he was referred to our institution.

**Figure 1:**
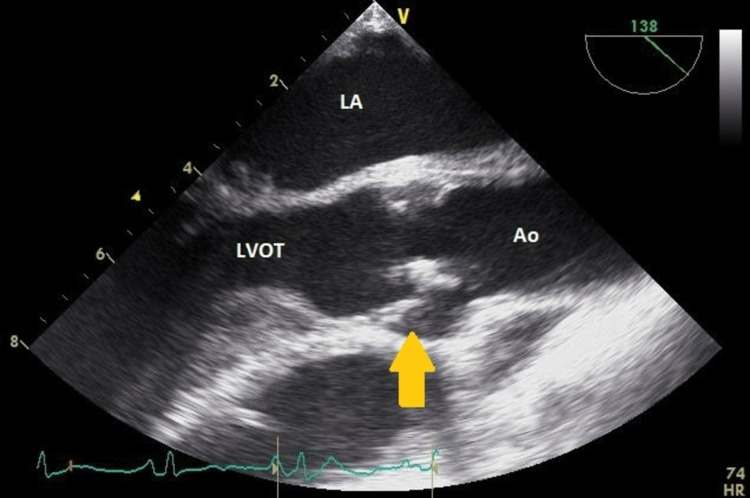
Thickened and restricted aortic valve leaflets (yellow arrow) due to leaflet infiltration by glycosaminoglycans are shown in the mid-esophageal long-axis LA = left atrium; LVOT = left ventricular outflow tract; Ao = aorta; RA = right atrium

A repeat preoperative echocardiogram was performed at our institution according to protocol. The aortic valve leaflets were severely thickened and calcified. The aortic valve was trileaflet and severely stenotic with a peak aortic transvalvular velocity of 4.6m/sec. The peak and mean transvalvular gradients were 85 and 57 mmHg, respectively, and the AVA was 0.8cm2 (0.5cm2/m2) using the continuity equation; these results were consistent with severe aortic stenosis (Figure 1). There was left ventricular hypertrophy and the ejection fraction was 55%.

**Figure 2:**
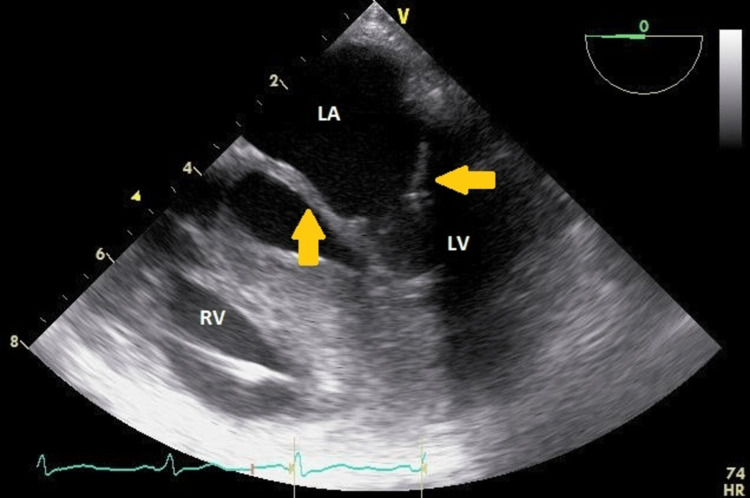
Thickened mitral valve leaflets (yellow arrow) due to leaflet infiltration by GAGs seen with doming and restriction during diastole in a modified mid-esophageal 5-chamber view. LA = left atrium; LV = left ventricle; RV = right ventricle; Ao = aorta

The echo also revealed moderate mitral stenosis with restricted opening and doming of the calcified mitral valve leaflets and trivial mitral valve regurgitation. The mean mitral valve gradient was 10mmHg with a heart rate of 84/min. The mitral area calculated by pressure half-time was 1.99cm2/m2 (Figure 2).

Anesthesia induction and perioperative management

**Figure 3:**
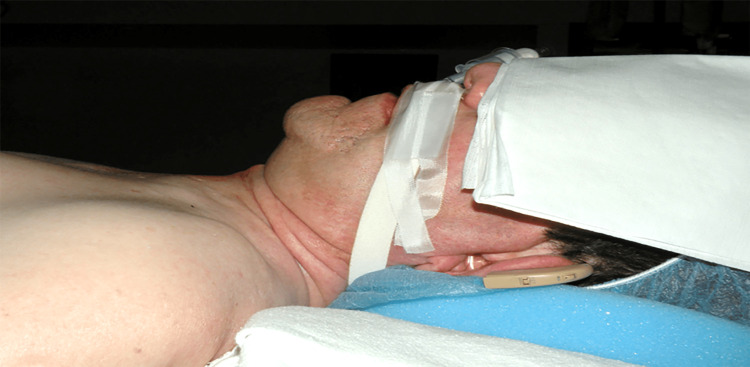
Facial deformity, small mouth open, short thyromental distance and expected anterior larynx

This patient had cervical spine immobility due to C1-3 laminectomy, short thyromental distance, a significant limitation of his temporomandibular joint, small mouth opening, macroglossia, and is considered a Mallampati class IV airway (Figure 3).

Because his airway anatomy provided difficult conditions for masking and endotracheal intubation using direct laryngoscopy, an awake tracheal intubation was performed. Nebulized and viscous lidocaine was used to topicalize the oropharynx, fentanyl and midazolam were given for sedation. While the patient was breathing spontaneously, fiberoptic bronchoscopy was used to pass an endotracheal tube through the cords. A 6.5mm endotracheal tube was attempted initially but excessive resistance was encountered; thus fiberoptic bronchoscopic tracheal intubation was reattempted with a smaller (6.0mm) endotracheal tube which was successful. During anesthesia induction and intubation, surgical standby was available in case we were unable to secure the airway and an emergency tracheostomy was needed. After the airway was secured, the patient was quickly induced with etomidate and fentanyl. Anesthesia was maintained with isoflurane and rocuronium was given for muscle relaxation. The patient required low-dose inotropic and vasopressor support with epinephrine and norepinephrine infusions after separation from cardiopulmonary bypass (CPB). The patient was transferred to the intensive care unit in stable condition.

Intraoperative transesophageal echocardiography (TEE)

Intraoperative TEE performed prior to CPB demonstrated normal left ventricular size and function with a mild reduction in right ventricular function. The patient’s native mitral valve demonstrated thickened leaflets with doming, restricted opening and 1+ mitral regurgitation. A mean mitral transvalvular gradient of 6mmHg with a heart rate of 70/min (normal sinus rhythm) was measured as consistent with moderate mitral stenosis. The native aortic valve demonstrated severe aortic valve stenosis caused by doming and restricted opening of a trileaflet valve. A peak transvalvular velocity of 3.9m/sec and peak and mean transvalvular gradient of 60 and 41 mmHg respectively were measured. The aortic valve area was calculated to be 0.5 cm2 using the continuity equation. The valve annular size was 22 mm and the sinotubular junction measured 3.2 cm. Moderate aortic regurgitation due to restricted motion and doming was also found.

Operative technique

Through a standard median sternotomy, the patient was fully heparinized and aortic and bicaval cannulation was performed. Myocardial protection was performed with an induction dose of antegrade cardioplegia delivered via the root and subsequent intermittent doses of ostial cold blood cardioplegia. His coronary sinus was too small to allow the placement of a retrograde cardioplegia cannula. An oblique aortotomy was performed at the sinotubular junction and the aortic valve was exposed. There was severe thickening and calcification of all three leaflets which were excised. The aortic valve was sized to a 21mm On-X mechanical valve. The Sondergaard’s groove was dissected and the left atrium was opened. The mitral valve was inspected and due to subvalvular and leaflet thickening, we did not feel repair was a suitable option. The anterior leaflet was excised and the posterior leaflet with choardae tendinae was preserved, and the valve was replaced with a 25mm On-X mechanical valve using 3-0 polypropylene continuous suture technique. Once the prosthesis was seated in position, attention was turned to the aortic valve which was then replaced with a 21mm On-X mechanical valve with 3-0 polypropylene continuous sutures. Routine procedures for cardioplegic arrest, subsequent defibrillation, and separation from CPB were followed.

Operative findings

**Figure 4:**
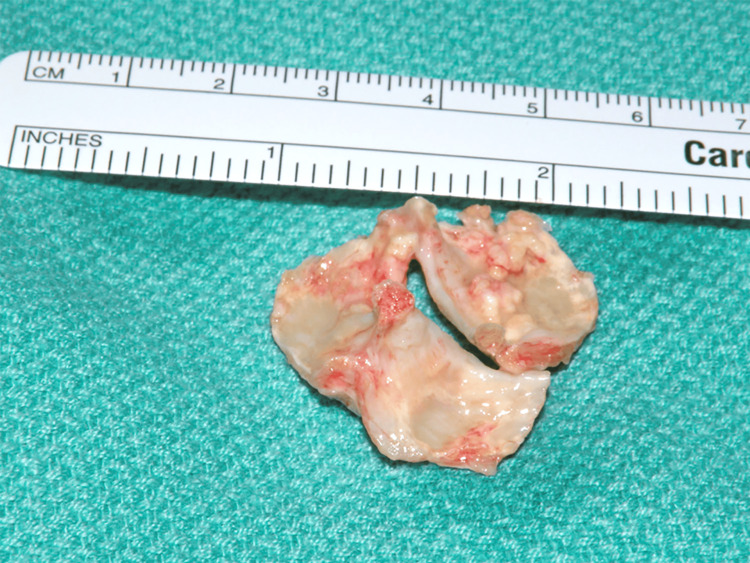
Excised aortic valve; severe thickening and calcification of all three cusps

The patient's aortic valve was trileaflet with severe thickening and calcification of all three leaflets (Figure 4).

**Figure 5:**
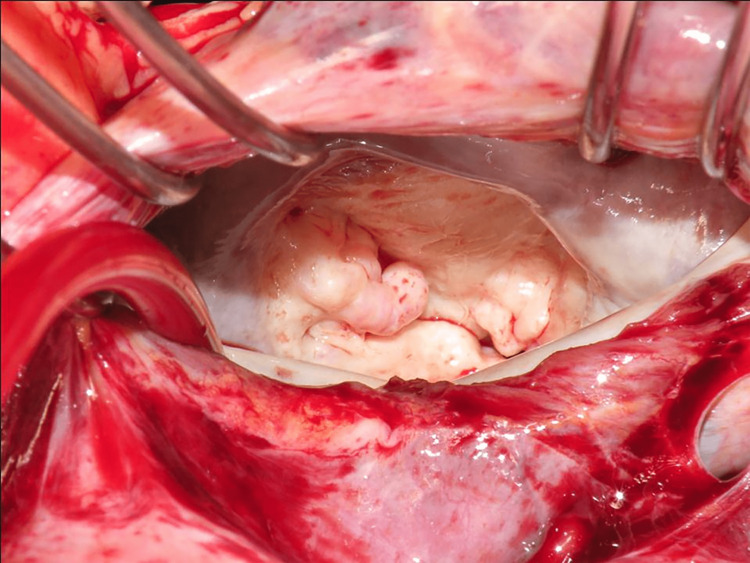
en face surgeon’s view of the mitral valve. Both mitral leaflets and the subvalvular apparatus including all of the chordae tendineae were thickened and fused

**Figure 6:**
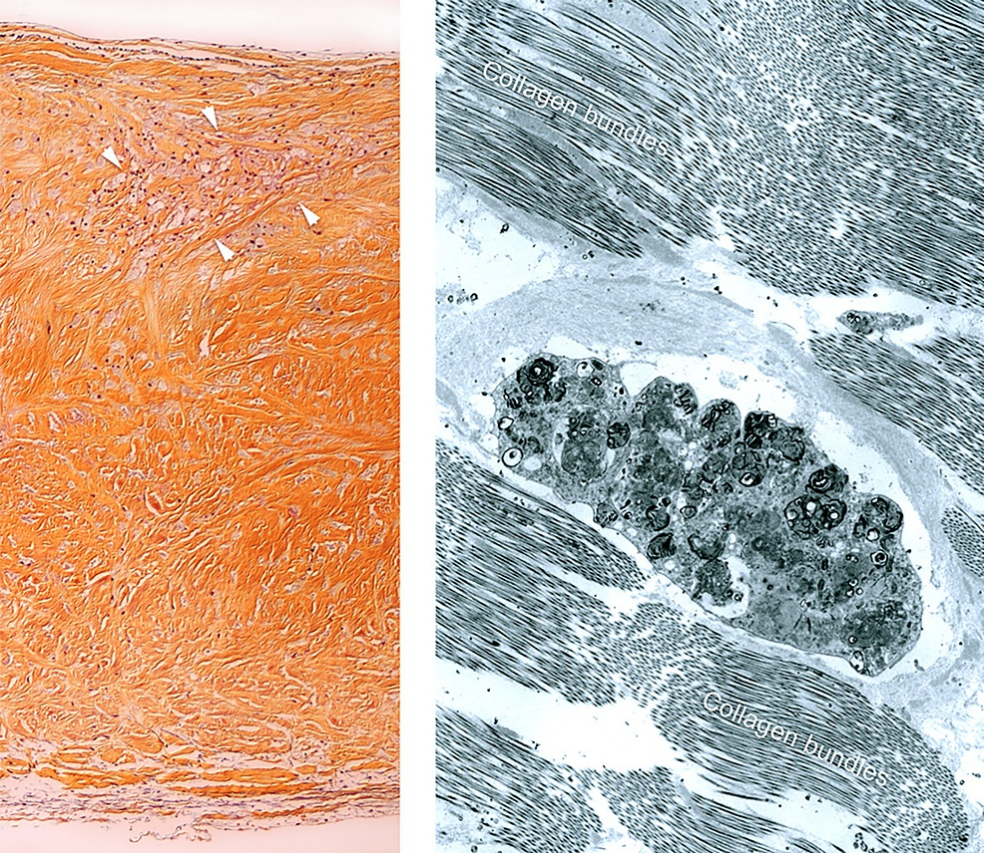
a) Light microscopy shows a transmural section of the aortic valve. There is dense fibrous tissue that has effaced the trilaminar architecture of the leaflet. This leaflet shows fibrosis replacing the spongiosa layer. The arrow heads show an area of “foamy” macrophages forming a nest. The foamy nature of the macrophages is due to accumulation of secondary lysosomes and mucopolysaccharides. (Movat pentachrome, X 200) b) Transmission electron microscopy shows dense collagen bundles flanking one macrophage from an area near the light microscopic section. The macrophage shows secondary lysosomes with lamellar bodies and amorphous dense bodies typically seen in mucopolysaccharidoses. (Uranyl acetate – Lead citrate, X 15,000) Inclusions show disruption and some-times absence of limiting membranes.

He also had what appeared to be the same degenerative process involving his mitral valve with thickening of the mitral leaflets. The mitral annulus was small. The anterior and posterior mitral leaflets were thickened and there was fusion of chordae tendinae (Figure 5). Light microscopy (Figure 6A) and electron microscopy (Figure 6B) of the excised valves showed dense fibrosis and "foamy macrophages" due to accumulation of secondary lysosomes and mucopolysaccharides respectively.

Intraoperative and hospital course and follow-up

TEE after CPB demonstrated normal left and right ventricular function with a well-seated prosthetic valve in the mitral position with a mean gradient of 4mm Hg. A prosthetic valve was also seen in the aortic position without any paravalvular leak, maximum velocity of 1.7m/sec, and peak and mean gradient of 11 and 5mm Hg, respectively. The patient did well postoperatively and the trachea was extubated uneventfully. He required prolonged chest tubes for air leak which resolved. An echocardiogram performed prior to discharge revealed normally functioning valve prostheses at both aortic and mitral positions. He was easily anticoagulated with warfarin.

## Discussion

MPS II is a variable, progressive, multisystem disorder. There are two variants; a severe form in which death supervenes by 10-15 yrs of age and a milder form in which patients survive into adulthood but death occurs between the ages of 20 and 30 years from cardiac or respiratory disease [9,10]. The hallmark is the intracellular deposition and increased urinary excretion of GAGs. Airway involvement, valvular cardiac disease, hearing impairment, carpal tunnel syndrome, and joint stiffness are common and can result in significant loss of function in both the mild and severe forms [9-11].

Airway management and tracheal intubation are technically challenging as the deposition of GAGs in the connective tissues are accompanied by an enlarged and thickened epiglottis, tonsillar hypertrophy, tongue enlargement, supraglottic narrowing, and a narrow and flattened trachea. This patient also had temporomandibular joint immobility which contributed to difficult airway management. This obstructive anatomy and physiology can lead to sleep apnea and airway obstruction [4,5,12]. As the disease progresses, the respiratory system is further compromised by pulmonary restriction secondary to the effects of the disease on the thoracic skeleton.

Compressive myelopathy results from vertebral dislocation complicated by the thickening of dural and ligamentous structures [13]. The most prominent features in the cervical spine are the thickening of the retro-odontoid tissue leading to canal stenosis and cord compression. Magnetic resonance imaging (MRI) scans of the craniocervical junction have shown deposition of GAGs around the tip of the odontoid process [9,14] and cervical laminectomy might become necessary in the presence of cord compression [15]. Our patient had undergone cervical laminectomy due to symptoms of compressive myelopathy.

Upper and lower respiratory tract infections are common. Because of these underlying airway and lung problems combined with facial dysmorphism, airway management can be very complex [9]. To address this concern for difficult airway management, we performed awake fiberoptic intubation with two attending anesthesiologists. As a potential rescue strategy, surgical standby for an emergency tracheostomy was available till the process of intubation was successfully accomplished. The equipment and personnel for performing emergency cricothyroidotomy were readily available so that it could be performed quickly and we also had the perfusionist in the room with the cannulas and equipment available to establish cardiopulmonary bypass if emergency mechanical circulatory support was required. Suzuki and colleagues discussed perioperative airway management while reporting a case of aortic valve replacement in Hunter syndrome and reiterated that awake intubation is helpful in these patients [16]. They advocated an approach emphasizing preoperative multidisciplinary airway assessment and simulation. Computed tomogram of the chest and neck is a valuable tool in the planning strategy. While our patient did not have a pre-operative CT of the chest we have modified our workup to include both a CT of the chest and neck going forward in this subset of patients. We emphasize the importance of echocardiography preparedness in a patient with left-sided valvular stenosis because of the added risk of rapid irreversible hemodynamic collapse. 

Cardiac disease in Hunter syndrome presents with valvular involvement due to the intracellular accumulation of GAGs which causes cellular enlargement, resulting in thickened, calcified, and loss of function of the tissue involved. Left-sided valves are predominantly affected and the presenting features are due to aortic stenosis, mitral stenosis or regurgitation, or a combination of both [17]. All four valves have been noted to be involved [18]. Heparin-dermatan, chondroitin, and keratin-sulfate GAGs are normal components of cardiac valves. Accumulation of GAGs is a pathologic hallmark of cardiac involvement in mucopolysaccharidosis, resulting in changes in the mechanical properties of the tissue and subsequent hemodynamic derangements. Failure of the maintenance of integrity in the extracellular matrix is becoming recognized and an important part of the pathogenesis of other valvular and vascular degenerative diseases [1,19,20]. Due to the potential for continued deposition of GAGs of the native valves which can compromise repair and lead to continued degeneration, valve replacement with mechanical valves is a suitable option.

Cardiac involvement in Hunter syndrome emerges at an early age and is progressive. Nearly two-thirds of the patients in the report by Kampmann et al. exhibited important cardiac signs and symptoms [21].

Wraith and colleagues reported that 57% of patients with Hunter syndrome have cardiac valve disease [9]. Mitral and aortic valves are more likely to be affected than right-sided heart valves. Bhattacharya et al. reported a case of mitral valve replacement for mitral stenosis and noted that mitral stenosis is less prevalent than mitral regurgitation in Hunter's syndrome [22]. Our patient had evidence of doming that can be seen in mitral regurgitation but the main mode of failure was mitral stenosis with thickened leaflets and thickened and shortened subvalvular apparatus seen in long-standing disease. Mitral stenosis is not unexpected in the advanced stages of Hunter’s syndrome but is less well known. Antoniou et al. have similarly reported a case wherein they had to use a 21mm mechanical aortic valve in a reversed position due to the small mitral orifice, an innovative approach to a difficult situation in the operating room [10]. Fortunately, we were able to fit a 25mm mechanical valve for our patient, but it would help to have smaller valve sizes available.

With the advent of transcatheter aortic valve replacement (TAVR) as a new standard therapy for aortic stenosis general anesthesia and intubation may be avoidable, but TAVR would have only allowed for treatment of one valve lesion and, moreover, the valve implanted are tissue valves which degenerate faster in younger patients. His mitral stenosis was quite significant to be left untreated. Transcatheter approaches to mitral stenosis are very limited and in a patient like this with a small left ventricular chamber and small valve orifices, this approach would be contraindicated even in an investigational setting. Double valve replacement in these patients has not been reported but multiple valve involvement should be considered an important component of the pathology. Most reports have used mechanical prostheses for replacing valves [10,22]. Avoiding repeat surgery inherent with the use of tissue valves or valve repair is an important consideration in these patients with progressive chronic disease due to deposition of GAGs. 

Recombinant human iduronate-2-sulfatase (idursulfase) has become available for the treatment of patients with MPS II. Enzyme replacement therapy (ERT) has the potential to help these patients provided that it is started early in the course of the disease [9,23]. Idursulfase was the first therapy to specifically address the underlying cause of the disease and in conjunction with appropriate supportive care remains a cornerstone of the management of patients with MPS II today. However current data on ERT with idursulfase suggest a limited therapeutic effect on aspects of the disease such as tracheal deformities, joint stiffness, bone deformities, and hearing loss [24].

## Conclusions

In conclusion, the management of patients with Hunter syndrome undergoing valve replacement surgery is complex and needs comprehensive team management. Successful strategies to manage the airway need to be formulated with rescue plans in case of difficulty. Valvular heart disease is typically managed by mechanical valve replacement due to severe tissue degeneration, which could involve multiple valves.
